# The impact of core self-evaluation on social anxiety among male nursing interns: the mediating role of self-acceptance

**DOI:** 10.3389/fpsyg.2025.1544629

**Published:** 2025-10-14

**Authors:** Fengxia Wang, Yufan Wang, Qihao Yang, Qiu Ju, Jinyan Li, Meng Liu, Yuxiao Wang

**Affiliations:** Pingdingshan University, Pingdingshan, China

**Keywords:** male nursing students, core self-evaluation, social anxiety, self-acceptance, mediating role

## Abstract

**Objective:**

This study aimed to examine the mediating role of self-acceptance in the relationship between core self-evaluation and social anxiety. Specifically, it aimed to investigate the effects of core self-evaluation and the impact of self-acceptance on social anxiety among male nursing students during their internship.

**Methods:**

A purposive sampling method was used to select a sample of 149 male nursing students from 8 tertiary hospitals in Henan Province. A questionnaire survey was conducted using the Core Self-Esteem Scale and the Social Anxiety Scale. The Scale of Self-Acceptance was also administered, and the data were statistically analyzed using SPSS software with the Process plug-in.

**Results:**

The results indicated a significant positive correlation between core self-evaluation and self-acceptance (*r* = 0.486), as well as a significant negative correlation between core self-evaluation and social anxiety (*r* = −0.559). Additionally, a significant negative correlation was observed between self-acceptance and social anxiety (*r* = −0.457). The direct effect of core self-evaluation on social anxiety was −0.584, while the indirect effect via self-acceptance was −0.157, resulting in a total effect size of −0.741 and a mediating effect size of 21.19%.

**Conclusion:**

The findings indicate that self-acceptance partially mediates the relationship between core self-evaluation and social anxiety among male nursing students during their internship.

## 1 Introduction

Social anxiety, characterized by feelings of nervousness, often emerges during daily interactions. It is recognized as a psychological disorder frequently associated with avoidance behaviors, blushing, and impaired expression, collectively posing significant challenges to engaging in typical social activities (Tabak et al., 2022). Nurses are particularly susceptible to social anxiety in the workplace due to the complex challenges they face when interacting with diverse patients (Majid et al., 2020). Nursing students in internship programs are especially vulnerable to anxiety, primarily due to their limited clinical experience. Notably, male nursing interns constitute a high-risk group, influenced by traditional gender norms, social discrimination, and other societal factors. Consequently, their job satisfaction and professional identity are progressively diminished (Russell and Topham, 2012). The exacerbation of these issues adversely affects both the physical functioning and mental health of male nursing students (Bentley et al., 2016; Koyuncu et al., 2019).

The National Health Commission has emphasized the importance of addressing nurses’ mental health. Research indicates that self-acceptance, defined as refraining from disapproving oneself based on personal abilities or the evaluations of others, forms the foundation of mental health. Social anxiety arises from a combination of internal and external factors. External factors include social disapproval (Haidl et al., 2019), while internal factors comprise personality traits (Tabak et al., 2022) and self-acceptance (Tabak et al., 2022; Liu et al., 2023). Furthermore, Haidl et al. (2019) demonstrated that social anxiety results from an individual’s fear of others’ perceptions and uncertainty regarding self-evaluation. Core self-evaluation refers to an individual’s assessment derived from domains such as personal competence and self-worth (Zou et al., 2022) and is considered the basis for other evaluations. Low core self-evaluation can hinder interpersonal relationship management, which is closely linked to social anxiety. Several studies suggest that core self-evaluation may serve as a direct predictor of social anxiety and also influences it indirectly through coping mechanisms and narrative disorders (Luo et al., 2024).

It is important to note that research examining the relationships among social anxiety, self-acceptance, and core self-appraisal is limited, particularly among male nursing interns (Joshanloo, 2022; Park and Shin, 2023; Single et al., 2022). The present study aimed to assess the levels of social anxiety in this population and to further explore the interplay between social anxiety, core self-appraisal, and self-acceptance. Additionally, an analysis of self-acceptance in relation to core self-appraisal and social anxiety was conducted, which may inform interventions targeting social anxiety in male nursing students.

## 2 Research subjects and methods

### 2.1 Subjects

A purposive sampling method was used to select male nursing students participating in internships at eight tertiary hospitals in Henan Province, China, from August to September 2024. Inclusion criteria included an internship duration of at least 3 months, provision of informed consent, and voluntary participation. Of the 160 distributed questionnaires, 149 valid responses were collected, yielding a validity rate of 93.13%. The average age of the male nursing interns was 21.57 ± 2.84 years, with 67 (44.97%) holding a college degree, 76 (51.00%) a bachelor’s degree, and 6 (4.03%) a postgraduate degree.

### 2.2 Research instruments

#### 2.2.1 Interaction anxious scale (IAS)

This study used the Social Anxiety Scale (IAS) developed by Leary in 1983 (Leary et al., 1983) and revised by Peng in 2004. The scale consists of 15 items scored on a 1–5 scale, with total scores ranging from 15 to 75; higher scores indicate more severe social anxiety. Items 3, 6, 10, and 15 are reverse-scored. The scale demonstrated good reliability, with a Cronbach’s *α* of 0.81 (Peng et al., 2004).

#### 2.2.2 Core self-evaluation scale (CSES)

In this study, the Core Self-Evaluation Scale (CSES), developed by Judge et al. (2003) and revised by Du et al. (2012), was used. The scale consists of 10 items scored on a 1–5 scale, with total scores ranging from 10 to 50; higher scores indicate higher levels of self-evaluation. The scale demonstrated good reliability, with a Cronbach’s *α* of 0.83 (Du et al., 2012).

#### 2.2.3 Self-acceptance questionnaire

The Self-Acceptance Questionnaire, developed by Cong and Gao (1999), was used in this study. The questionnaire consists of 16 items rated on a 1–4 scale ranging from “very much the same” to “very much the opposite,” with total scores ranging from 16 to 64. Higher scores indicate higher levels of self-acceptance among participants. The scale demonstrated excellent reliability, with a Cronbach’s α of 0.9347 (Cong and Gao, 1999).

### 2.3 Data collection and ethical considerations

Approval for this study was obtained from the Board of Pingdingshan University. The purpose, significance, and confidentiality of the survey were explained in the guidance section of the questionnaire. The questionnaire was digitized as a QR code using Questionnaire Star and distributed via the social platforms WeChat and QQ.

### 2.4 Statistical processing

SPSS 22.0 was used to perform descriptive and Pearson’s correlation analyses on core self-evaluation, social anxiety, and self-acceptance among male nursing interns. The PROCESS plug-in was used to examine the mediating role of self-acceptance between core self-evaluation and social anxiety. A *p*-value of <0.05 was considered statistically significant.

## 3 Results

### 3.1 Status and correlation of core self-evaluation, self-acceptance, and social anxiety in male nursing interns

As shown in Table 1, the mean core self-evaluation score of male nursing interns was 36.181 ± 3.367, the mean self-acceptance score was 41.007 ± 4.635, and the mean social anxiety score was 47.725 ± 4.465. A moderately positive correlation was observed between core self-evaluation and self-acceptance (*r* = 0.486), while core self-evaluation and social anxiety were moderately negatively correlated (*r* = −0.559). Additionally, self-acceptance and social anxiety showed a moderately negative correlation (*r* = −0.457).

**Table 1:** Status and correlation of core self-evaluation, self-acceptance, and social anxiety among male nursing interns.

Variable	Score	Core self-evaluation	Self-acceptance	Social anxiety
Core self-evaluation	36.181 ± 3.367	1		
Self-acceptance	41.007 ± 4.635	0.486^**^	1	
Social anxiety	47.725 ± 4.465	−0.559^**^	−0.457^**^	1

**indicates *p* < 0.05.

### 3.2 The effect of core self-evaluation on social anxiety among male nursing students

As shown in Table 1, significant correlations exist between the variables. To explore the mediating role of self-acceptance between core self-evaluation and social anxiety among male nursing students, the Bootstrap method proposed by Hayes (2013) was used with 5,000 resamples. A 95% confidence interval that does not include 0 indicates a significant mediating effect (Cai et al., 2022). In this analysis, core self-evaluation was used as the independent variable, social anxiety as the dependent variable, and self-acceptance as the mediator.

**Figure 1:**
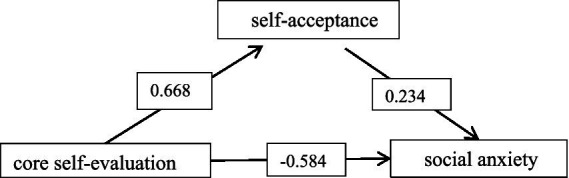
Modeling of core self-evaluation, social anxiety, and self-acceptance among male nursing interns.

The results of the regression analysis indicated that core self-evaluation significantly and negatively predicted social anxiety (*B* = −0.741, *p* < 0.001). After including self-acceptance in the regression model, core self-evaluation continued to significantly and negatively predict social anxiety (*B* = −0.584, *p* < 0.001). Mediation analysis showed that the direct effect of core self-evaluation on social anxiety was −0.584, with a 95% confidence interval of −0.783 to −0.386, while the indirect effect via self-acceptance was −0.157, with a 95% confidence interval of −0.305 to −0.001. The total effect was −0.741, with a 95% confidence interval of −0.929 to −0.561, resulting in a mediating effect size of 21.19% (−0.157/−0.741 × 100%) (see Figure 1).

## 4 Discussion

### 4.1 Current status and correlation analysis of core self-evaluation, self-acceptance, and social anxiety in male nursing interns

As shown in Table 1, the core self-evaluation score of male nursing interns (36.181 ± 3.367) was lower than that reported by Chen et al. (2022), indicating a relatively low level of core self-evaluation among male nursing students. The self-acceptance score (41.007 ± 4.635) was significantly lower than that reported by Park and Shin (2023), suggesting a lower level of self-acceptance. The social anxiety score (47.725 ± 4.465) was higher than that reported by Cao et al. (2023), indicating an elevated level of social anxiety among male nursing students.

Core self-evaluation was significantly positively correlated with self-acceptance (*r* = 0.486) and significantly negatively correlated with social anxiety (*r* = −0.559), while self-acceptance was significantly negatively correlated with social anxiety (*r* = −0.457). Core self-evaluation (Xu et al., 2018) serves as a foundation for mental health and is essential for enhancing job satisfaction. Lower core self-evaluation scores among male nursing students may be associated with a lack of professional identity and uncertainty regarding career goals. Luo et al. (2024) suggested that core self-evaluation is influenced not only by personal traits but also by external environmental factors. This study involved male nursing students in their internship phase, newly entering clinical practice. Social biases and traditional beliefs often discourage acceptance of male students performing procedures like catheterization and skin preparation (Turan et al., 2019), which can lower core self-evaluation, reduce self-efficacy, and create doubts about professional competence. As a result, self-acceptance declines, social anxiety rises, and clinical interactions become more confusing, creating a cycle where heightened anxiety further diminishes self-evaluation and confidence (Ruan et al., 2023).

### 4.2 The influence of core self-evaluation on social anxiety among male nursing interns: the mediating role of self-acceptance

As shown in Tables 2, 3, core self-evaluation significantly and negatively predicted social anxiety (*B* = −0.741, *p* < 0.01). Self-acceptance played a partial mediating role between core self-evaluation and social anxiety (B = −0.234, *p* < 0.01). These results support the hypothesis, indicating that core self-evaluation among male nursing students not only directly predicts social anxiety but also indirectly influences it through self-acceptance, aligning with the findings of Dong (2023) and Chen (2024).

**Table 2:** Regression analysis of core self-evaluation, self-acceptance, and social anxiety of male nursing students in practice.

Dependent variable	Independent variable	*R*	*R^2^*	*F*	*B*	*t*
Social anxiety	Core self-evaluation	0.558	0.312	66.727	−0.741	−8.169
Self-acceptance	Core self-evaluation	0.485	0.235	45.356	0.668	6.735
Social anxiety	Core self-evaluation	0.598	0.357	40.601	−0.584	−5.806
	Self-acceptance				−0.234	−3.204

**Table 3:** Decomposition of mediating effects of core self-evaluation, self-acceptance, and social anxiety among male nursing interns.

Effect	Effect value	BootSE	BootCI limit	BootCI lower limit	Mediated effect value
Total effect	−0.741	0.091	−0.561	−0.929	
Direct effect	−0.584	0.101	−0.386	−0.783	
Mediated effect of self-acceptance	−0.157	0.076	−0.001	−0.305	21.19%

Male nursing students with higher levels of core self-evaluation are more likely to affirm their abilities and value in clinical practice, maintain a positive attitude toward their performance, and adjust their mindset promptly when faced with negative feedback or doubt. Consequently, they exhibit lower levels of social anxiety. Numerous studies have shown that individuals with higher social anxiety are more sensitive to negative evaluations from others and tend to attribute these to personal inadequacies (Leichsenring and Leweke, 2017; Danforth et al., 2018). Reichenberger and Blechert (2018) further indicated that such individuals not only perceive negative evaluations more readily but also feel anxious about positive feedback, fearing that others may raise expectations based on their good performance (Rooney et al., 2023). Therefore, it is essential for instructors, peers, patients, and family members to guide male nursing students in recognizing their strengths, evaluating themselves comprehensively, forming positive self-assessments, and providing greater understanding and support. This approach can foster an optimistic attitude, enhance core self-evaluation, and alleviate social anxiety during the internship period.

According to the model, the mediating effect of self-acceptance between core self-evaluation and social anxiety was 21.19%, indicating that self-acceptance is a significant internal factor influencing social anxiety among male nursing students, consistent with the findings of Liu et al. (2023). Numerous studies have shown that low self-acceptance stems from an inability to let go of past mistakes and a fear of making errors, which inhibits action (Muroni and Sudres, 2023; Plexico et al., 2019). For male nursing interns, low self-acceptance may result from concerns about making mistakes during clinical procedures and fears of disapproval or doubt from peers, instructors, patients, and families, hindering positive social interactions.

The internship period represents a transition from theoretical learning to clinical practice; therefore, it is crucial to enhance students’ mastery of basic knowledge, theory, and skills while developing non-intellectual competencies, such as emotional intelligence, to improve adaptation to interpersonal relationships and boost self-acceptance. Additionally, hospital managers should focus on cultivating management and research skills and guide students to explore diverse career paths. Finally, hospitals and educational institutions should highlight the influence of role models, such as Basang Dengzhu, the only male nurse in China awarded the “Nightingale Medal,” to strengthen professional identity and reduce social anxiety among male nursing students.

## 5 Conclusion

Core self-evaluation among male nursing interns can directly predict social anxiety and indirectly influence it through the mediating role of self-acceptance. Both educational institutions and hospitals can help reduce social anxiety in male nursing students by implementing interventions that enhance core self-evaluation and promote self-acceptance.

## Data Availability

The original contributions presented in the study are included in the article/supplementary material, further inquiries can be directed to the corresponding author.
